# Virtual reality motor sensing exercise in patients with Parkinson’s disease: a scoping review

**DOI:** 10.3389/fresc.2025.1630304

**Published:** 2025-07-31

**Authors:** Lulu Zou, Xiaoqing Chen, Sisi Lei, Qingwen Hu

**Affiliations:** ^1^ School of Information and Engineering, First Clinical Medical College, Wenzhou Medical University, Wenzhou, China; ^2^ Department 341, The First Affiliated Hospital, Wenzhou Medical University, Wenzhou, China

**Keywords:** virtual reality, exercise, Parkinson's, novel rehabilitation, scoping review

## Abstract

**Background:**

A scoping review of research on the application of virtual reality (VR) motor sensing exercises for patients with Parkinson's disease was conducted to identify the types of interventions, outcome indicators, and evaluation tools used and to assess the effectiveness of these exercises. The aim was also to provide a reference for future research in this area.

**Methods:**

The aim of this scoping review was to examine the current status of research into the application of somatosensory virtual reality exercise for patients with Parkinson's disease. We conducted a systematic search of the PubMed, Cochrane Library, Web of Science, and Embase databases. The search time frame was from the date the library was established until 19 April 2025, with the included literature being screened and summarised.

**Results:**

The majority of the included studies reported improved rehabilitation outcomes for participants, suggesting that VR is beneficial for the rehabilitation of patients with Parkinson's disease. A total of 2,327 articles were retrieved, comprising 10 randomised clinical trials, 3 class-experimental studies, and 1 mixed study involving a total of 470 patients with Parkinson's disease.

**Conclusion:**

This scoping review provides a basis for the application of virtual reality somatosensory exercise in elderly patients with Parkinson's disease and lays the groundwork for future research and clinical practice. However, large-scale, high-quality randomised controlled trials are still needed to verify the feasibility of virtual reality somatosensory exercise for Parkinson's patients and to inform the development of targeted exercise programmes for this patient group.

## Introduction

Parkinson's disease (PD) is a typical example of a neurodegenerative disease characterised by motor impairments, postural instability, and non-motor symptoms (including cognitive impairment and affective disorders). These symptoms lead to a progressive deterioration in motor function, an increased risk of falls, and a significant reduction in quality of life (1). Studies have shown that the global prevalence of PD is expected to reach 25.2 million cases by 2050, marking a 112% increase from the 2021 baseline year and underscoring the severity of the disease burden (2). In the field of rehabilitation medicine, intervention strategies for balance dysfunction in patients with Parkinson’s disease remain iterative. Although traditional physical therapy is evidence-based, its limitations have led to the development of innovative interventions, such as virtual reality (VR) technology, which creates computer-generated artificial environments for the construction of clinically adapted, immersive rehabilitation scenarios (3). VR's core technological advantages include (1) a dynamically adjustable treatment parameter configuration system to support adaptation to the heterogeneous rehabilitation needs of patients with PD (4), (2) a multi-sensory integrated training environment that enhances neural plasticity through audiovisual and vestibular sensory coupled feedback (5), (3) a real-time biofeedback mechanism based on motion capture that effectively strengthens the efficiency of motor skill acquisition (6), and (4) high-dose, high-repeatability functional task simulation that overcomes the operational limitations of traditional training modes (7). In clinical practice, the VR system facilitates visual-motor integration via interactive interfaces, such as head-mounted displays (HMDs) and surround-screen projection (8). When combined with personalised rehabilitation programme generation algorithms, it can significantly enhance therapeutic adherence and patient initiative (9). Given the above technical characteristics, VR somatosensory intervention has become a cutting-edge area of PD neurorehabilitation research. This study uses the Scoping Review methodological framework to systematically review the application scenarios, intervention effects, and existing challenges of VR somatosensory movement in elderly patients with Parkinson's disease, with a view to providing a reference for future research and practice.

## Materials and methods

### Study design

We conducted a scoping review to address our objectives, beginning with a systematic search, screening, and integration of relevant studies now available on the use of virtual reality somatic movement in Parkinson's patients. This review is based on the framework for scoping reviews proposed by Arksey and O'Malley (10), with the following steps: (1) identification of the research objectives and research questions, (2) development of a literature search plan and retrieval of relevant literature, (3) identification of inclusion and exclusion criteria and screening of the literature, (4) evaluation of the quality of the literature, and (5) collation and summarising of results.

### Research question and eligibility criteria

Based on the objectives of this scoping review, we identified the following research questions: (1) What are the types of VR somatic movements for Parkinson's patients? (2) What are the outcome indicators and evaluation tools for VR somatic movement? (3) What are the challenges of VR somatosensory exercise in Parkinson's patients and implications for future research?

We included articles that met the following eligibility criteria:

• The study population (population, P) was pathologically clearly diagnosed as Parkinson's sufferers.
• The concept (concept, C) was patients who received virtual reality somatic movement therapy for the improvement of Parkinson's symptoms.
• Context (context, C) was the scenario in which the patient received the intervention, including receiving virtual reality somatosensory exercise during hospitalisation or in a home environment.

We excluded articles that met the following exclusion criteria:

• Non-English literature.
• Full text not available in English.
• Reviews or systematic evaluations, conferences, policies and guidelines, or research proposals.

### Search strategy

**Figure 1:**
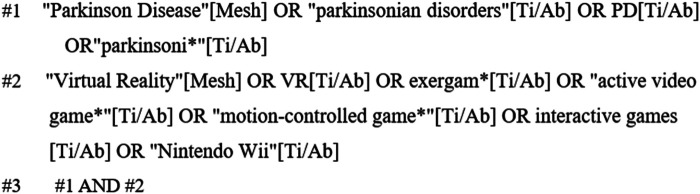
Search strategies of PubMed.

PubMed, Embase, Web of Science, and the Cochrane Library were systematically searched from the time of construction to 19 April 2025 by two researchers. The search terms were “Parkinson Disease, parkinsonian disorders, PD, parkinsoni*”, “Virtual Reality, VR, exergam*,active video game*, motion-controlled game*, interactive games, Nintendo Wii. interactive games,Nintendo Wii”. The search was conducted using a combination of grid subject terms and free words, and the references included in the literature were further tracked and checked. Taking PubMed as an example, the search formula is shown in Figure 1.

### Data management and extraction

#### Literature screening

Literature was imported into EndNote software to remove duplicates, and two researchers trained in evidence-based medicine independently screened the literature according to inclusion and exclusion criteria by reading the titles and abstracts and then reading the entire text for re-screening. In case of disputes or disagreements during the screening process, a third researcher was consulted and the final decision of inclusion was made.

#### Data extraction and analysis

Two researchers independently extracted data from the included literature and discussed with the third researcher in case of disagreement. Extracted information included author, publication date, country, study type, study population, sample size, mean age, intervention, intervention duration, and outcome indicator measurement tools.

## Result

### Search results

**Figure 2:**
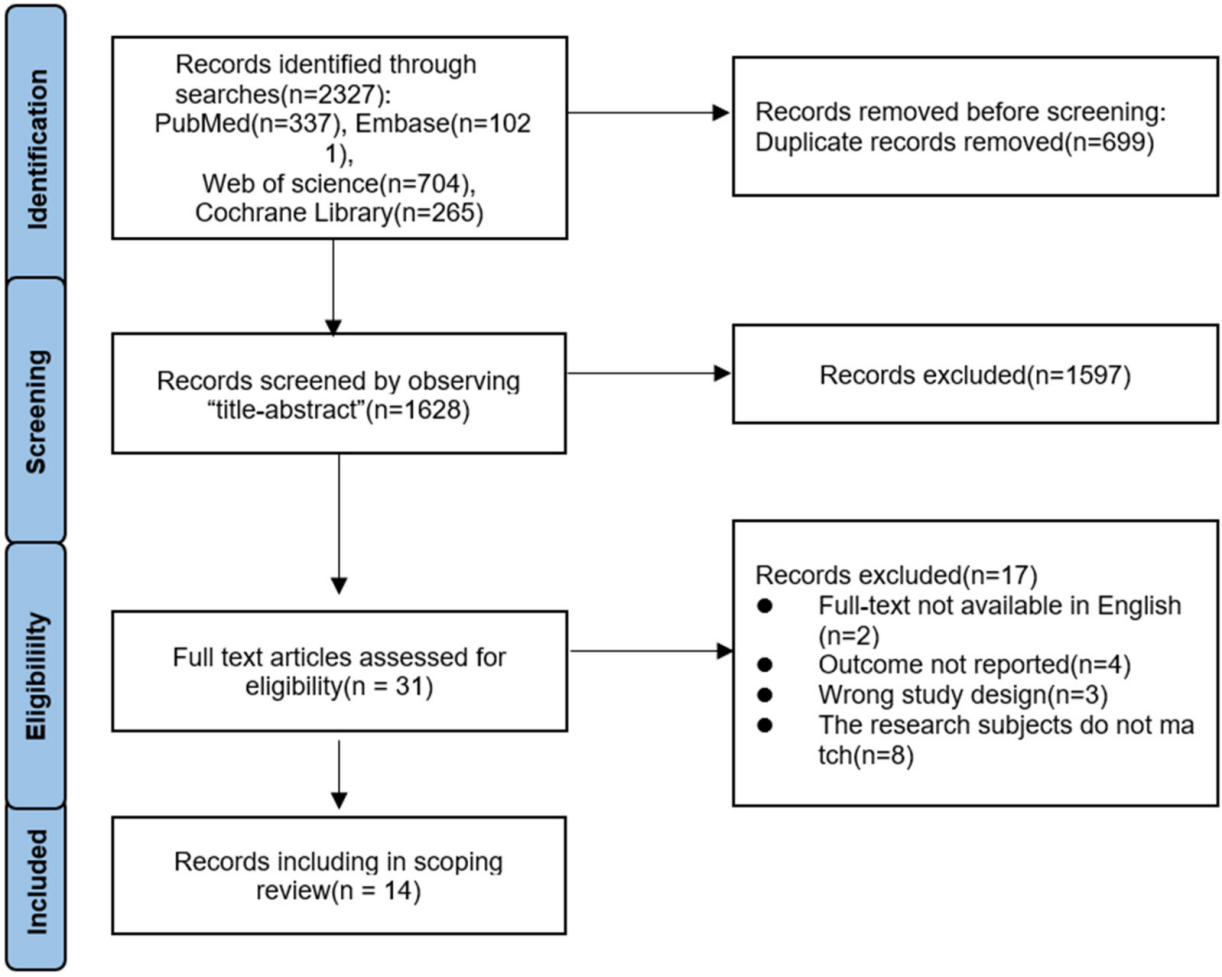
Flow diagram of the literature screening process.

A total of 2,327 articles were obtained from this literature search, with the breakup being 337 articles from PubMed, 1,021 articles from Embase, 704 articles from Web of Science, and 265 articles from Cochrane library. The literature was imported into endnote, 699 duplicates were removed, a total of 1,628 articles were screened according to the title and abstract, and 1,597 articles were excluded. The full text of 31 articles was assessed for suitability and 17 were excluded because they did not meet the inclusion criteria. Fourteen articles were finally included in this paper and the flowchart of literature screening is shown in Figure 2. The publication period was from 2020 to 2025, and of the 14 articles, 4 (11–14) were from Italy, 2 (15, 16) from Spain, 2 (17, 18) from Brazil, 2 (19, 20) from Pakistan, 1 (21) from the Republic of Korea, 2 (22) from China, 1 (23) from Turkey, and 1 (24) from the Czech Republic. Of these, 10 articles were randomised clinical trial (RCT) studies, 3 were experimental-like studies, and 1 was a mixed study. The sample size ranged from 6 to 49 patients. The details are given in Table 1.

**Table 1:** Characteristics of the included studies (*n* = 14).

Reference	Year	Country	Study design	Age (years) experimental/control	Population	Sample
Yun et al. (21)	2023	Republic of Korea	Quasi-experimental study	73.83 ± 6.09	Idiopathic PD	12
Yuan et al. (22)	2020	China	Quasi-experimental study	67.8 ± 5.5/66.5 ± 8.8	Mild-to-moderate PD	12/12
Sánchez-Herrera-Baeza et al. (15)	2020	Spain	Hybrid research	74.50 ± 4.72	PD	6
Rodriguez-Fuentes et al. (16)	2024	Spain	RCT	70.87 ± 6.67/70.59 ± 6.67	Idiopathic PD	30/22
Qayyum et al. (19)	2022	Pakistan	RCT	52.5	PD	8/8
Pullia et al. (11)	2023	Italy	RCT	64.5 ± 10.84/65.5 ± 10.36	PD	10/10
Nuvolini et al. (17)	2025	Brazil	RCT	62.74 ± 6.80/69.21 ± 7.82	Mild-to-moderate PD	19/19
Maranesi et al. (12)	2022	Italy	RCT	72.7 ± 6.3/75.5 ± 5.4	PD	16/14
Lombardi et al. (13)	2024	Italy	RCT	—	PD	24/24
Kashif et al. (20)	2024	Pakistan	RCT	63.20 ± 4.85/61.95 ± 4.62	Mild-to-moderate PD	20/20
Honzíková et al. (24)	2025	Czech Republic	Quasi-experimental study	64.2 ± 12.8	PD	19
Gulcan et al. (23)	2022	Turkey	RCT	61/60	PD	15/15
Goffredo et al. (14)	2023	Italy	RCT	67.8 ± 6.6/68.2 ± 5.8	PD	49/48
Da Silva et al. (18)	2023	Brazil	RCT	63.33 ± 6.46/68.00 ± 10.02	Mild-to-moderate PD	18/20
Reference	Intervening measure		Experimental/control	Duration	Outcomes	Tool
Yun et al. (21)	Virtual reality scenario interactive training (boxing and leg agility exercises)		—	For 2–3 times a week, 10 times, 30 min each time	①, ②, ③, ⑦	(a), (b), (c), (d), (e), (f)
Yuan et al. (22)	Physical exercise in virtual reality		—	For 6 weeks, three times a week	①, ②, ⑤	(a), (b), (c), (g), (h), (i)
Sánchez-Herrera-Baeza et al. (15)	Interactive games were conducted using the Oculus Rift 2 motion-sensing interactive device and the jumping motion controller to train the upper limb movement results + cognitive training		—	For 6 weeks, three times a week, 30 min each time	①, ③, ⑤	(C), (D), (E)
Rodriguez-Fuentes et al. (16)	Head-mounted virtual reality environments (such as simulating pedalling in different cities in Europe)		Conventional treatment plans include physical therapy (pedalling), cognitive training, etc.	For 12 weeks, twice a week, 25 min each time	①,②,③,⑦	(a), (b), (f), (j), (k), (l), (m)
Qayyum et al. (19)	Wii Fit, Wii Perfect Ten Balances, Wii-Ski, and Wii Cycling		The conventional treatment plan includes balance training, gait training, stretching, and muscle strength training	For 8 weeks, three times a week, 50 min each time	①, ②, ⑧	(a), (b)
Pullia et al. (11)	Training is conducted using the C-Mill system, featuring a head-mounted virtual reality environment combined with running training		Regular running	For 5 weeks, four times a week, 45 min each time	①, ②, ③, ④, ⑤, ⑥	(a), (b), (c), (n), (o), (r), (s), (k)
Nuvolini et al. (17)	Use motion-sensing interactive devices such as Microsoft Kinect 2.0 for motion-sensing interactive games		Conventional physiotherapy based on the central areas of the European guideline	For 7 weeks, twice a week, 60 min each time	②, ④	(b), (o), (t), (u), (v)
Maranesi et al. (12)	Remote game motion system		Conventional systemic treatment	For 5 weeks, twice a week, 50 min each time	①, ②, ③,④,⑤	(p), (q), (w), (x), (y)
Lombardi et al. (13)	Intensive treadmill training was conducted using the C-Mill system		Intensive treadmill training	For 8 weeks, three times a week	①, ②, ③, ④, ⑤, ⑥	(a), (d), (e), (p), (k), (n), (z)
Kashif et al. (20)	The Wii Fit project includes tennis, boxing, bowling, and kicking games		Conventional physical therapy	For 12 weeks, three times a week, 60 min each time	①, ②, ⑤	(a), (c), (l), (z)
Honzíková et al. (24)	Head-mounted virtual reality environment, exercising the upper and lower limbs		—	For 4 weeks, twice a week, 20 min each time	①, ②, ④, ⑥, ⑧	(b), (c), (k), (o), (v)
Gulcan et al. (23)	Training is conducted using the C-Mill system		Conventional physical therapy	For 6 weeks, three times a week, 60 min each time	①, ②, ③, ⑦	(a), (b), (c), (A)
Goffredo et al. (14)	Remote game movement system (movement for training lower limbs)		Structured conventional training	For 6–10 weeks, 30 times a week, 3–5 times	①, ②, ⑤	(a), (n), (t)
Da Silva et al. (18)	Use motion-sensing interactive devices such as Microsoft Kinect 2.0 for motion-sensing interactive games		Conventional physical therapy	For 7 weeks, twice a week, 60 min each time	①, ②, ⑤, ⑥	(a), (b), (j), (k), (A), (B)

RCT, randomised clinical trial; PD, Parkinson's disease.

Outcome measures: ① Balance, ② Gait, ③ Cognitive ability, ④ Fear of falling, ⑤ Physical activity, ⑥ Quality of life, ⑦ Feasibility, and ⑧ Depression or anxiety. Outcome indicator measurement tool: (a) UPDRS, Unified Parkinson's Disease Rating Scale; (b) TUG, Timed Up and Go Test; (c) BBS, Berg Balance Scale; (d) ST, Stroop Test; (e) Trail-Making Test; (f) SSQ, Simulator Sickness Questionnaire; (g) MFES, Modified Falls Efficacy Scale; (h) Multi-Directional Reach Test; (i) UST, Unipedal Stance Test; (j) FSTST, Five Sit-To-Stand Test; (k) PDQ-39, Parkinson's Disease Questionnaire; (l) SUS, System Usability Scale; (m) GEQ-Post Game, Game Experience Questionnaire; (n) 6MWT, 6 min Walking Test; (o) 10MWT, 10 m Walking Test; (p) POMA-Gait, Performance-Oriented Mobility Assessment-Gait; (q) SF-12, 12-Item Short Form Health Survey; (r) TS, Tinetti Scale; (s) FIM, Functional Independence Measure; (t) MoCA, Montreal Cognitive Assessment; (u) ST, single task; (v) DT, dual task; (w) MCS-12, Mental Component Score; (x) FES-I, Falls Efficacy Scale, International; (y) GAS, Goal Attainment Scaling; (z) FOG-Q, Freezing of Gait Questionnaire; (A) ABCS, Activities-Specific Balance Confidence Scale; (B) mini-BESTest; (C) CSQ-8, Client Satisfaction Questionnaire; (D) PPT, Purdue Pegboard Test; and (E) ARAT, Action Research Arm Test.

### The types of intervention for virtual reality motor sensing exercise

Types of VR somatic exercise include exercise in VR environments, telekinesis gaming systems, Wii Fit exercise, C-Mill system training, and somatic interactive games. Among the literature included in the analysis, seven studies (11, 13, 16, 21–24) explored exercise modalities in VR environments, specifically boxing and leg agility training based on interactive training in virtual reality scenarios, virtual reality sports workouts, and simulated cycling experiences in different cities in Europe through head-mounted devices. In addition, running training under the C-Mill system is an important component of exercise in VR environments, with the main goal of improving running speed, stride frequency, and directional control. Compared with traditional training, under C-Mill, a balanced perception of confidence can be ensured by its safety equipment and simple instructions for performing VR exercises, as shown by Pullia et al. (11). Two other studies (12, 14) focused on the Remote Motion Gaming System, and two studies (19, 20) dealt with Wii Fit sports, which cover seven different modes, namely Wii Perfect Ten Balances, Wii-Ski, Wii Cycling, Wii Tennis, Wii Boxing, Wii Bowling, and Wii Kicking Game. Finally, three studies (15, 17, 18) reported on the application of somatic interactive gaming, in which Oculus Rift 2 and Microsoft Kinect 2.0 somatic interactive devices were mainly used to achieve a diverse range of sports experiences.

### Outcome indicators and evaluation of virtual reality motor sensing exercise

The classification of outcome indicators of VR somatic movement includes four aspects: physical indicators, cognitive function, psychological indicators, and quality of life. (1) Physical indicators include balance, gait, and other content, covering Timed Up and Go (TUG) test (*n* = 9), Berg Balance Scale (BBS) (*n* = 6), Modified Falls Efficacy Scale (*n* = 1), Multi-Directional Reach Test (*n* = 1), Unipedal Stance Test (*n* = 1), Five Sit-to-Stand Test (*n* = 2), 6 min Walking Test (6MWT) (*n* = 3), 10 m Walking Test (10MWT) (*n* = 3), Performance-Oriented Mobility Assessment-Gait (POMA-gait) (*n* = 2), Tinetti Scale (TS) (*n* = 1), Functional Independence Measure (FIM) (*n* = 1), single task (*n* = 1), dual task (*n* = 2), falls efficacy scale, international (*n* = 1), Free (*n* = 1), and international (*n* = 1), Freezing of Gait Questionnaire (FOG-Q) (*n* = 2), Mini-BESTest (*n* = 1), Purdue pegboard test (*n* = 1), and Action Research Arm Test (*n* = 1). (2) The psychological component assesses anxiety and depression mental component score (*n* = 1), including Activities-Specific Balance Confidence Scale (ABCS) (*n* = 2). (3) The cognitive component, which encompasses overall cognitive functioning, including motor speed, attention, memory, and verbal ability, is the main component of the Unified Parkinson's Disease Questionnaire (PDQ) (*n* = 2), Unified Parkinson's Disease Rating Scale (UPDRS) (*n* = 10), Stroop Test (*n* = 2), Trail-making (*n* = 2), Simulator Sickness Questionnaire (*n* = 2), System Usability Scale (*n* = 2), Game Experience Questionnaire (*n* = 1), 12-Item Short Form Health Survey (*n* = 1), Montreal Cognitive Assessment (*n* = 2), Goal Attainment Scaling (*n* = 1), and client satisfaction questionnaire (*n* = 1). (4) Quality-of-life aspects were assessed primarily through the Parkinson's Disease Questionnaire (*n* = 4).

### Effectiveness of VR for PD

Effective outcomes include positive results, results with significant improvement, and negative results (no significant improvement, no statistical analysis, or inconsistent results). In 14 articles, 92.8% (13/14) reported outcomes compared with the pre-intervention group, and 85.7% (12/14) reported outcomes compared with the control group. The reported results showed that in the 13 articles comparing the intervention group, all reported good results compared with the pre-intervention group, including improvements in balance, gait, cognitive function, physical activity, quality of life, feasibility, and psychological aspects; specifically the TUG, BBS scores, Stroop test, Tinetti test, MDS-UPDRS, PDQ-39 scores, 6MWT, Mini-BESTest, 10MWT, ABCS scores, FES-I, MCS-12, FIM, TS, and POMA scales showed significant improvements. In the control group, 41.7% (5/12) reported good outcomes compared with the earlier outcomes, including improvements in balance, gait, quality of life, compliance, and cognitive function, specifically in the 6MWT, BBS, TS, UPDRS-III, FIM, Mini-BESTest, PDQ-39, and ABCS.

### The application effect of virtual reality motor sensing exercise

Research data show that VR somatosensory training modalities produce significant effects on functional rehabilitation in patients with PD, see Table 2 for specific improvement indicators. Specifically, 13 studies (11–16, 18–24) showed that exercising in a VR environment, the Remote Motion Game System, Wii Fit exercise, and somatosensory interactive games could improve the balance of patients, as shown in the study by Maranesi et al. (12)'s study utilized the MCS-12 scale and demonstrated a statistically significant result in the psychological field (MCS-12: 17.1 ± 0.4 vs. 16.5 ± 0.4, *P* = 0.034). Thirteen studies (11–14, 16–24) indicated that the above modalities could also improve the gait of patients. Studies such as Qayyum et al. (19) showed that the average dynamic gait index score improved from 14.12 to 16.21 points. Maranesi et al. (12) showed that the gait score improved from 9.7 ± 0.8 to 11.4 ± 0.2 after intervention (*P* = 0.003). Seven studies (11–13, 15, 16, 21, 23) studies showed that the Remote Motion Gaming System improved cognitive functioning. Five studies (11–13, 17, 24) showed that the Remote Motion Gaming System and the Somatic Interactive Game reduced patients` fear of falling, Maranesi et al. (12)'s study utilized the MCS-12 scale and demonstrated a statistically significant result in the psychological field (MCS-12: 17.1 ± 0.4 vs. 16.5 ± 0.4, *P* = 0.034). Eight studies (11–15, 18, 20, 22) showed that the Remote Motion Gaming System, Wii Fit exercise, Scenario-Based Interactive Training, and Somatic Interactive Game improved physical mobility. Sánchez-Herrera-Baeza et al. (15) observed significant improvements in strength (*P* = 0.028), fine motor skills (*P* = 0.026–0.028), overall coordination and dexterity, and speed of movement (*P* = 0.039) on the affected side. Four studies (11, 13, 18, 24) showed that somatosensory interactive games could improve patients’ quality of life. Honzíková et al. (24) found significant improvements in motor function in the experimental group in the PDQ-39 questionnaire (*P* = 0.027; R = 0.51). Three studies (16, 21, 23) reported the feasibility of somatosensory interactive games for the rehabilitation of Parkinson's patients. One study (19) reported that Wii Fit exercise can improve the mood of patients.

**Table 2:** Effectiveness of VR for PD.

Reference	Main results reported	
	Compared with pre-intervention	Compared with control
Yun et al. (21)	Following intervention, the number of steps taken during the TUG test under physical dual-task conditions was significantly reduced (*P* = 0.045). BBS scores improved from 49.5 to 51.00, *P* = 0.047. Stroop colour-word test scores improved from 37.50 ± 11.94 to 43.33 ± 10.22, *P* = 0.003. There were no significant changes in UPDRS, Trail-Making Test, or digit span test scores	—
Yuan et al. (22)	BBS scores improved from 3.1 ± 4.9 to 9.9 ± 7.29.9, *P* < 0.0001	—
Sánchez-Herrera-Baeza et al. (15)	Significant improvements were observed in strength (*P* = 0.028), fine motor skills (*P* = 0.026–0.028), overall coordination and dexterity, and speed of movement (*P* = 0.039) on the affected side, with excellent compliance (100%)	—
Rodriguez-Fuentes et al. (16)	Timed Up and Go test (*P* = 0.028), Tinetti test (*P* = 0.046), PDQ-39 score (*P* = 0.035), and MDS-UPDRS score (*P* = 0.001, no dropouts, high compliance)	No significant differences (*P* > 0.05)
Qayyum et al. (19)	Following intervention, the average difference in the UPDRS score decreased from 87.25 to 81.12, and the average score on the Dynamic Gait Index increased from 14.12 to 16.21. The study found that movement games have a significant improvement effect on balance and gait in patients with Parkinson's disease	No significant differences (*P* > 0.05)
Pullia et al. (11)	6MWT (*P* < 0.0005, ES = 0.93), TUG (*P* < 0.03, ES = 0.33), BBS (*P* < 0.006, ES = 1.16), TS (*P* < 0.002, ES = 0.89), FES-I (*P* < 0.03, ES = 0.46), UPDRS-III (*P* < 0.002, ES = 0.48), and FIM (*P* < 0.004, ES = 1.13)	6MWT (*P* < 0.01, ES = 0.41), BBS (*P* < 0.004, ES = 0.50), TS (*P* < 0.01, ES = 0.59), UPDRS-III (*P* < 0.01, ES = 0.60), and FIM (*P* < 0.005, ES = 0.38)
Nuvolini et al. (17)	TUG and ST time in all participants [F(1.49; 52.23) = 5.786; *P* = 0.010]. There was a significant improvement in gait stability and functional mobility. FGA, 10MWT in ST, 10MWT in DT, MoCA (*P* > 0.5)	Were similar to the results of the VR intervention group
Maranesi et al. (12)	POMA total: 24.6 ± 0.9 vs. 25.9 ± 0.7, *P* = 0.010. There were also improvements in gait characteristics (9.7 ± 0.8 vs. 11.4 ± 0.2, *P* = 0.003) and balance function (13.8 ± 0.5 vs. 14.7 ± 0.4, *P* = 0.004). In the psychological domain, MCS-12 (17.1 ± 0.4 vs. 16.5 ± 0.4, *P* = 0.034)	Balance function 12.4 ± 0.7 vs. 13.5 ± 0.8, *P* = 0.017
Lombardi et al. (13)	Not reported	No significant differences
Kashif et al. (20)	There was a significant improvement in motor function after intervention: 33.95 ± 3.501 vs. 17.20 ± 9.451, *P*-value = 0.001. The BBS score was 37.15 ± 3.437 vs. 50.10 ± 4.897 (*P* = 0.019). ABCS scores were 57.95 ± 4.629 vs. 78.59 ± 6.386, with a statistically significant difference (*P* < 0.05). UPDRS II scores were 25.20 ± 3.036 vs. 15.30 ± 2.364, *P* = 0.000	No significant differences (*P* > 0.05)
Honzíková et al. (24)	Following intervention, the time required for the 10MWT was significantly reduced (*P* = 0.006; *r* = 0.63) and the TUG (*P* < 0.001; R = 0.80). BBS scores improved significantly after treatment (*P* = 0.016; R = 0.55). In the PDQ-39 questionnaire, the experimental group showed significant improvements in motor function (*P* = 0.027; R = 0.51) and emotional health (*P* = 0.011; R = 0.58)	No significant differences (*P* > 0.05)
Gulcan et al. (23)	UPDRS-III, postural measurement, BBS, ABC, spatiotemporal gait parameters, and TUG improved in the study group (*P* < 0.05)	BBS, ABC, and only spatial gait parameters (except step width) improved in the control group (*P* < 0.05)
Goffredo et al. (14)	Mini-BESTest total score, *P* < 0.001, 6MWT, *P* < 0.001, Cohen's d: 0.653	Mini-BESTest total score, *P* < 0.001, MDS-UPDRS motor section *P* < 0.05
Da Silva et al. (18)	Mini-BESTest, PDQ-39, UPDRS-III, compliance rates were all greater than 75%	Mini-BESTest, PDQ-39, UPDRS-III, compliance rate all greater than 75%

UPDRS, Unified Parkinson's Disease Rating scale; TUG, Timed Up and Go Test; BBS, Berg Balance Scale; ST, Stroop Test; PDQ-39, Parkinson's Disease Questionnaire; 6MWT, 6 min Walking Test; 10MWT, 10 m Walking Test; POMA-gait, Performance-Oriented Mobility Assessment-Gait; TS, Tinetti Scale; MoCA, Montreal Cognitive Assessment; ABCS, Activities-Specific Balance Confidence Scale; DT, dual task; ES, effect size; FGA, functional gait assessment.

## Discussion

### Virtual reality motor sensing exercise has feasibility in PD

The feasibility of VR somatosensory exercise has been demonstrated in a number of clinical studies, which have clearly confirmed its applicability in patients with PD. Among the included studies, two mentioned patient compliance, reporting high compliance, no dropouts during the study, and compliance rates of >75% in both the control and the experimental groups. Furthermore, none of these studies reported any adverse events. Qayyum et al. (19) and other scholars have demonstrated that patients with PD show a high level of motivation to participate in scenario-simulated exercise game training, and that the efficiency of the acquisition of manipulative skills is significant. This finding serves to substantiate the hypothesis that the VR somatosensory training mode is both enjoyable and acceptable to users. A review of epidemiological data indicates that the number of patients diagnosed with PD in China has reached 2.6 million cases, representing approximately 50% of the global total. Projections indicate that this figure is likely to exceed 5 million by the year 2030. In this context, there is an urgent need to promote the clinical application of VR technology in the field of cognitive function enhancement and movement disorder intervention for patients with PD. With regard to the evaluation of effectiveness, systematic studies have demonstrated that VR somatosensory exercise can exert a multidimensional positive effect on patients, encompassing the core dimensions of cognitive function improvement, somatic mobility enhancement, optimisation of mental health indicators, and significant improvement in quality of life. This underscores the strategic importance of VR somatosensory motion technology in the development of neurological rehabilitation systems, and its large-scale implementation is anticipated to yield innovative solutions for the optimisation of medical resources.

### The problems of virtual reality motor sensing exercise in patients with PD

Although VR has great application value in exploring the pathogenesis and rehabilitation of PD patients, it also faces many challenges. (1) There is a wide variety of training devices and games based on VR technology, such as C-Mill, Xbox Kinect, Wii Fit VR, and other VR rehabilitation training systems specifically tailored for patients with PD. However, as of now, there is no research that clearly indicates which types of training devices, games, and training programmes are more suitable for patients’ rehabilitation training or how to set the intensity of each type of device and training method to prevent injuries to patients with PD due to over-exercise. (2) There are various forms of VR physical exercise, and the outcome indicators and evaluation tools used for different types of exercise are not entirely consistent. Therefore, it is necessary to further improve the relevant content and develop corresponding outcome indicators and evaluation tools based on different sports. (3) The intervention time of the literature included in this study ranged from 4 to 12 weeks, which is a relatively short period of time and lacks validation of the effect of exercise function maintenance. Moreover, the minimum sample size of the included literature was 6 and the maximum was 49, which is generally small and lacks a large sample size. Long-term randomised controlled trials as well as multi-sample trials are needed in the future to further assess the effectiveness of VR in the rehabilitation of patients with PD. (4) Most of the patients in the literature included in this study were over 60 years’ old on average. When applying VR technology to the elderly, technical difficulties may be encountered, leading to technical anxiety in patients, which, in turn, reduces compliance. At the same time, the use of VR technology in the treatment of elderly patients with PD requires a significant investment in human and equipment costs. Therefore, when promoting the application of VR technology to patients with PD on a large scale, not only do we need to develop individualised supporting programmes for elderly patients, but we also need corresponding policy support, such as the inclusion of VR rehabilitation in medical insurance.

### Implications for future practice and research

The application of VR somatosensory exercise in Parkinson's patients is a promising area of research, as VR uses computer-generated virtual environments to deeply immerse the user and cleverly integrates motivational strategies into the training process in the form of “gamification,” which greatly enhances adherence to highly repetitive, high-intensity functional training (25). This is a highly innovative health rehabilitation tool for patients with PD, and initial results have been achieved so far. In the future, the field could be explored in depth in the following key areas: (1) Establishing clear guidelines: To accurately determine the training intensity of various rehabilitation training devices, a large number of high-quality, multi-centre randomised controlled trials need to be conducted. For example, exercise difficulty should be set based on UPDRS scores, and training programmes more suitable for patients with PD patients should be developed. In addition, outcome measurement and evaluation tools corresponding to various exercise programmes need further refinement. (2) Obtaining evidence-based medical evidence of long-term efficacy: long-term follow-up studies on patients with PD participating in VR sensory training (lasting >12 weeks) to verify the impact of VR on maintaining motor function and clarify the long-term efficacy of VR for such patients should be conducted. (3) Utilising single-case randomised controlled trials, also known as N-of-1 trials: while RCT studies provide population-level evidence, they lack the ability to account for individual differences. Given the significant variability in symptom fluctuations and drug responses observed in individuals diagnosed with Parkinson's disease, conducting a single randomised controlled trial is highly necessary. Based on the baseline characteristics of patients, a distinction must be made between tremor-dominant PD, postural instability-dominant PD, and patients’ cognitive status, and then the corresponding rehabilitation programme must be selected; patients with symptoms such as balance disorders, TUG improvement, and freezing of gait must be prioritised, with freezing of gait patients prioritised for FOG-Q scoring. Rehabilitation training methods more acceptable to older patients must be developed, corresponding policy support must be actively sought, and rehabilitation budgets effectively reduced.

## Conclusion

Existing research has proven the feasibility and effectiveness of applying virtual reality sensory training to patients with Parkinson's disease, demonstrating its significant clinical value and practical application potential. Future recommendations include integrating domestic and international research findings to optimise training intensity adjustment mechanisms, intervention programme design, and multidimensional evaluation systems for VR-based sensory training in a systematic way. Further research is also recommended to determine the most appropriate VR training intensity and to explore its sustained efficacy for patients with PD through long-term longitudinal studies. Furthermore, randomised controlled single-case trials should be conducted to develop personalised intervention pathways for patients with PD based on individual differences, developing elderly-friendly interfaces, thereby promoting the comprehensive application of VR sensory training in the management of neurodegenerative diseases.
